# Reference data on reaction time and aging using the Nintendo Wii Balance Board: A cross-sectional study of 354 subjects from 20 to 99 years of age

**DOI:** 10.1371/journal.pone.0189598

**Published:** 2017-12-29

**Authors:** Andreas W. Blomkvist, Fredrik Eika, Martin T. Rahbek, Karin D. Eikhof, Mette D. Hansen, Malene Søndergaard, Jesper Ryg, Stig Andersen, Martin G. Jørgensen

**Affiliations:** 1 Department of Geriatric and Internal Medicine, Aalborg University Hospital, Aalborg, Denmark; 2 Department of Medicine, Østfold Hospital, Kalnes, Norway; 3 Department of Geriatric Medicine, Odense University Hospital, Odense, Denmark; 4 Emergency Clinic, Aalborg University Hospital, Aalborg, Denmark; 5 Department of Health Science and Technology, Aalborg University, Aalborg, Denmark; 6 Institute of Clinical Research, University of Southern Denmark, Odense, Denmark; 7 Department of Clinical Medicine, Aalborg University, Aalborg, Denmark; University of Toronto, CANADA

## Abstract

**Background:**

Falls among older adults is one of the major public health challenges facing the rapidly changing demography. The valid assessment of reaction time (RT) and other well-documented risk factors for falls are mainly restricted to specialized clinics due to the equipment needed. The Nintendo Wii Balance Board has the potential to be a multi-modal test and intervention instrument for these risk factors, however, reference data are lacking.

**Objective:**

To provide RT reference data and to characterize the age-related changes in RT measured by the Nintendo Wii Balance Board.

**Method:**

Healthy participants were recruited at various locations and their RT in hands and feet were tested by six assessors using the Nintendo Wii Balance Board. Reference data were analysed and presented in age-groups, while the age-related change in RT was tested and characterized with linear regression models.

**Results:**

354 participants between 20 and 99 years of age were tested. For both hands and feet, mean RT and its variation increased with age. There was a statistically significant non-linear increase in RT with age. The averaged difference between male and female was significant, with males being faster than females for both hands and feet. The averaged difference between dominant and non-dominant side was non-significant.

**Conclusion:**

This study reported reference data with percentiles for a new promising method for reliably testing RT. The RT data were consistent with previously known effects of age and gender on RT.

## Introduction

“The world is facing a situation without precedent: We soon will have more older people than children and more people at extreme old age than ever before” [1]. Such is the beginning of the WHO report *Global Health and Aging*, highlighting the rapidly changing demography of the world population. It stresses the importance of a coordinated research effort to develop, improve, and provide innovative solutions to the upcoming challenges in public health [2]. Due to its high frequency and huge consequences in terms of health care service expenditure, morbidity, and mortality, falls among older people is one of these major challenges [3–5]. Tripping is the most commonly reported cause of falling [6], however, it is most often the result of an interaction between identifiable environmental hazards and the increased individual susceptibility [3]. Accordingly, home hazard assessment and modification together with individual multifactorial risk assessment and intervention are the mainstay of fall prevention in older adults [7].

A well-documented risk factor of falls is increased reaction time (RT) in both upper and lower extremities [8–10]. This is thought to be because RT reflects neurocognitive processing speed and functioning. For instance, prospective studies have identified visual processing speed to independently predict injurious falls [11,12] and a recent review found that intraindividual RT variability has a greater predictive utility for falls than mean RT [13]. RT is also responsive to exercise interventions [14,15] and has been extensively tested in various populations, most commonly by measuring the time between a visual stimulus and hitting a response button [16]. However, due to the use of expensive laboratory equipment [17–20], predominantly available at universities and hospitals, a clinically feasible cost-effective test of RT has yet to be developed [10]. Some methods have shown promising results [10, 20], while others have proven to be unreliable [21]. One promising new method is with the use of a standard Nintendo Wii Balance Board (WBB). With game-based assesment [22] or custom software, such as FysioMeter [23], developed at the geriatric department at Aalborg University Hospital, the WBB could become a relevant clinical tool for fall risk assessment. Firstly, the WBB is a widely available, inexpensive, and portable system. Secondly, it has demonstrated good within-day and between-day reproducibility (intraclass correlation coefficient 0.76–0.87), and it is able to differentiate between young and older adults in both upper and lower limb RT tests [24]. Thirdly, WBB has shown similar or better reproducibility results for other clinically relevant parameters, such as isometric handgrip strength [25], balance assessment [26,27], and uni- [28] and bilateral [29] lower limb strength. Finally, it has successfully been used as intervention tool for enhancing balance control in older adults [30].

However, studies using the WBB indicate that individual results on relevant clinical parameters are not directly interchangeable with other instruments, e.g. the Jamar handdynamometer [25] or stationary isometric dynamometers [29]. Moreover, the assessment of RT is susceptible to different experimental protocols and set-ups, and the result of one method is rarely interchangeable with another. Before using WBB to measure RT in a clinical setting, there is a need for valid reference data. So far, only smaller studies investigating the validity of WBB have been published. Therefore, the aims of this study are (1) to provide reference data on RT in a larger heterogeneous study-population and (2) to characterize the age-related changes in RT in both males and females.

## Method

### Study-design and recruitment

To simulate common fall-risk assessment, we tested RT in conjunction with balance and strength assessments in a cross-sectional study using six different assessors. All measurements were performed during a single home visit. Participants were recruited at various locations (e.g. malls, local communities, university campus, and hospital staff) during the spring and summer of 2016 in Denmark (Aalborg and Odense) and Norway (Oslo and Ålesund). Participants were eligible for inclusion if they were at least 20 years old and reported good self-perceived health. Participants were excluded if they had clear cognitive problems (not being able to name current year or the national capital), could not stand unsupported for 30 seconds, had significant neuromuscular disease (e.g. sequelae after stroke or Parkinson’s disease), or musculoskeletal disease (i.e. recent (< 6 months) orthopedic surgery or bone fracture, muscular dystrophy, or polymyositis rheumatica). Lastly, individuals with alloplastic surgery within the last two years were excluded. Participants gave oral consent and the study was approved by the ethics committee of the North Jutland Region, Denmark.

### Equipment and software

The WBB is a rectangular-shaped platform, which has four uniaxial vertical strain gauge force transducers, one in each corner. From the WBB data were streamed using Bluetooth technology to a personal computer and into FysioMeter (Bronderslev, Demark). The FysioMeter software obtained data from the transducers in the WBB by four channels of 16-bit digital data samples at approximately 100 Hz. The data were filtered using a 4th order Butterworth filter with a cut-off frequency of 20 Hz.

### Overall experimental procedure

Prior to any measurements, the assessors collected information on age, gender, weight, height, hand and leg dominance (i.e. “with which side do you prefer to throw/kick a ball?”), smoking status (i.e. never, current, or prior), and number of prescription drugs used daily. Furthermore, participants’ physical activity level was assessed from 1 (least active) to 4 (most active) for work (if applicable) and leisure hours, as done in the Copenhagen City Heart Study [31].

Harmonization of the experimental procedure was secured between the six assessors (AWB and FE (medical students), MTR (medical doctor), KDE and MS (nurses), and MDH (physiotherapist)) at the Department of Geriatrics, Aalborg University Hospital before the study was initiated. To minimize systematic bias, each assessor recruited participants for every pre-defined age category: 20–29, 30–39, 40–49, 50–59, 60–69, 70–79, and 80+ years. The assessment of RT was done after the assessment of postural balance, but before the measurement of muscle strength. The experimental procedure for the assessment of postural balance and muscle strength are fully described in the cited reproducibility studies [25,29,32].

### Reaction time procedure

Each participant started with the lower limb RT measurement by standing with the feet shoulder width apart, 5 cm behind the WBB, which was placed on the floor in front of the participant. The WBB is 5 cm x 30 cm x 50 cm, meaning that the participants stepping motion had to exceed the 5 cm height. In preparation for the RT tests, participants were instructed to bear equal weight on each leg (i.e. not to have one leg more ready for stepping). The computer screen visualized a virtual WBB, and was placed on a table 80–100 cm in front of the eyes of the participant. When the test started, a green visual stimulus appeared at a random side (left or right side of the board) at a random time (1–4 seconds) on the virtual WBB board. Participants were instructed beforehand that this indicated which side to tap the board as quickly as possible with the appropriate foot. When the participant tapped the board, the internal timer would stop and the time from stimulus to the registered hit was recorded in milliseconds (ms). The next attempt was initiated immediately after the previous one, for a total of seven assessments. For the first six, the software was designed to include three attempts for each side (left and right) at a random order. The last and seventh attempt served as a dummy to prevent the participant from anticipating which side the stimulus would appear. Importantly, the full seven-attempt session was stopped and repeated if the participant during one of the assessments either lost focus on the screen, missed the board during hitting, or used the non-designated side to hit the board.

For the upper limb RT assessment, the participant was seated in a chair with arms resting and fist clenched 5 cm in front of the WBB. As with the lower limb RT assessment, a green visual stimulus appeared on a random side at a random time interval between 1 and 4 seconds for seven attempts. The participant stopped the internal timer by hitting the board on the designated side as fast as possible. For the same indications as for the lower limb RT measurement, the seven-attempt session was stopped and repeated.

### Statistical analysis

All statistical analyses were performed using SPSS (version 24). All results are given with mean ± standard deviation (SD) for normally distributed data (Shapiro-Wilk) or median with interquartile range for non-normal distributions. From the FysioMeter software, four variables of mean RT were extracted from each participant: RT hands for dominant and non-dominant side (RTH-D and RTH-ND, respectively), and RT feet for dominant and non-dominant side (RTF-D and RTF-ND, respectively). Variables were divided into gender and age groups (20–29, 30–39, 40–49, 50–59, 60–69, 70–79, and 80+) and assessed for outliers using the outlier labelling rule [33]. With the exception of extreme outliers, indicative of measurement errors, outliers were winsorized [33]. Also, for each gender and age-group, the 10, 25, 75, and 90 percentiles were extracted. We used one-sample t-test and independent t-test to test for significant difference between the mean RT for each side (dominant and non-dominant) and gender, respectively. Non-normal data were tested with non-parametric tests, i.e. Wilcoxon Signed-Rank test and Mann-Whitney U test.

To investigate the age-related changes in RT, four linear regression models were calculated using the mean RT for hands and feet for each gender, i.e. mean RT as dependent variable and age as independent variable. Assumption of linearity and homoscedastisticity was assessed with the standardized residuals plotted against predicted values, while the assumption of normal distributed errors and autocorrelations was assessed with the histogram of residuals and Durbin-Watson test (accepted value between 1.5 and 2.5), respectively [34]. Violations of linearity or homoscedasticity were corrected with transformations. The presence of non-linear relationships were statistically tested with hierarchical multiple regression using a quadratic model, i.e. adding the age squared as an independent variable to our linear regression models.

## Results

A total of 354 participants, age 20–99 years, were recruited and tested. Participant characteristics and reaction time results (in percentiles) are given in Tables 1 and 2, respectively.

**Table 1:** Anthropometric data.

Age group (years)	Gender (number)	Age (years)	BMI (kg/m^2^)	Medicine (number)	Smoking (N;C+P)%	Physical activity level work	Physical activity level leisure
**20–29**	Male 22	24.5±2.5	25.1±3.1	0 [0–1]	86;14	2 [2–3]	3 [3–4]
	Female 36	24.7±2.2	22.1±2.5	0 [0–1]	86;14	2 [2–3]	3 [2–3]
**30–39**	Male 15	33.3±2.4	25.7±5.0	0 [0–0]	73;27	2 [2–3]	3 [2–3]
	Female 30	34.0±2.3	26.3±5.9	0 [0–1]	80;20	2 [2–3]	2 [2–3]
**40–49**	Male 20	44.7±2.9	27.3±4.4	0 [0–0]	55;45	2 [2–3]	3 [2–4]
	Female 21	45.6±3.0	27.1±4.8	0 [0–0]	66;34	3 [2–3]	3 [2–4]
**50–59**	Male 16	54.9±3.3	26.0±3.3	0 [0–1]	62;38	2 [2–3]	3 [2–3]
	Female 30	54.4±2.9	25.7±4.0	1 [0–2]	53;47	2 [1–3]	2 [2–3]
**60–69**	Male 19	65.3±2.3	29.1±6.4	0 [0–4]	42;58	2 [1–3]	3 [2–3]
	Female 35	65.6±2.9	26.7±4.8	1 [0–2]	60;40	2 [1–3]	3 [2–3]
**70–79**	Male 32	73.5±2.8	27.2±3.5	1 [0–3]	44;56	*	3 [2–3]
	Female 33	73.6±2.8	26.6±4.1	1 [1–5]	64;36	*	2 [2–3]
**80+**	Male 20	85.6±4.1	26.5±3.1	3 [0–6]	35;65	*	2 [1–3]
	Female 25	85.6±4.0	25.0±6.6	3 [1–5]	52;48	*	3 [2–3]

Medicine refers to number of drugs used daily. Smoking is divided into never (N) and current (C) or prior (P), and given in percentages. Physical activity at work and during leisure time is reported in medians from 1 (least active) to 4 (most active).

* An insignificant number of participants were working (i.e. most participants were fully retired).

**Table 2:** Reaction time results (in percentiles) for male and female divided into age groups.

Age group	RTH-D Percentiles Median [10, 25, 75, 90]	RTH-ND Percentiles Median [10, 25, 75, 90]	RTF-D Percentiles Median [10, 25, 75, 90]	RTF-ND Percentiles Median [10, 25, 75, 90]
**20–29**				
Male	460 [416, 436, 519, 552]	483 [424, 454, 557, 591]	632 [532, 581, 668, 768]	629 [582, 603, 651, 698]
Female	521 [436, 477, 555, 615]	514 [454, 485, 544, 621]	685 [585, 622, 751, 786]	694 [609, 657, 733, 764]
**30–39**				
Male	491 [390, 432, 544, 560]	469 [403, 433, 534, 562]	639 [500, 525, 674, 724]	640 [493, 539, 693, 802]
Female	518 [444, 477, 588, 660]	538 [461, 499, 590, 619]	695 [595, 632, 762, 847]	672 [601, 624, 768, 860]
**40–49**				
Male	533 [445, 481, 579, 715]	524 [426, 451, 575, 684]	702 [576, 631, 735, 836]	682 [570, 599, 770, 923]
Female	557 [472, 514, 639, 661]	524 [461, 497, 616, 671]	731 [627, 688, 815, 831]	750 [650, 693, 824, 876]
**50–59**				
Male	547 [423, 506, 599, 640]	559 [426, 522, 586, 705]	754 [578, 635, 826, 899]	748 [625, 653, 801, 940]
Female	592 [480, 539, 627, 728]	605 [479, 524, 653, 701]	789 [694, 743, 836, 902]	808 [696, 717, 869, 900]
**60–69**				
Male	617 [466, 553, 651, 725]	602 [452, 550, 627, 684]	788 [687, 751, 909, 1031]	830 [699, 744, 879, 1009]
Female	671 [542, 608, 794, 1007]	655 [534, 581, 784, 866]	877 [743, 770, 964, 1154]	835 [739, 786, 949, 1171]
**70–79**				
Male	650 [499, 571, 769, 941]	707 [547, 609, 769, 935]	931 [719, 797, 1025, 1213]	903 [762, 830, 981, 1162]
Female	683 [541, 601, 842, 1089]	725 [587, 642, 859, 953]	1004 [783, 835, 1150, 1421]	949 [778, 850, 1068, 1388]
**80+**				
Male	707 [525, 630, 839, 1131]	759 [548, 588, 873, 1124]	1005 [697, 863, 1119, 1443]	1035 [791, 833, 1232, 1262]
Female	893 [564, 670, 1017, 1260]	836 [615, 668, 1085, 1179]	1200 [802, 876, 1458, 1603]	1217 [824, 898, 1338, 1675]

RTH-D, RTH-ND, RTF-D and RTF-ND denotes reaction time for the dominant hands, non-dominant hands, dominant feet and non-dominant feet, respectively. Values presented are in milliseconds.

There were three and five extreme outliers for RT hands and feet, respectively. As expected, all four variables of reaction time data were positively skewed. Histogram for each of the four variables are available as supplementary material online. In general, the mean or median age for each gender was comparable in every age-group and our study population reported a fairly high level of physical activity during leisure time. Additionally, in the age-groups 60–69, 70–79 and 80+ there were about 20 percentage points more females compared to males reported never smoking. For all four variables of RT, there was an increase in RT and variation with higher age group (Table 2). Using Mann-Whitney U-test, the difference between male and female was statistically significant for both hands (Z = -2.71, p = 0.007) and feet (Z = -2.08, p = 0.037), with males being faster than females. The approximated difference, assessed with the independent t-test, was -50 milliseconds (95% CI: -84;-16) and -55 milliseconds (95% CI: -101;-9) for hands and feet, respectively. Turning to the difference between dominant and non-dominant side, Wilcoxon Signed-Rank test was non-significant for hands (Z = -1.35, p = 0.178) and feet (Z = -1.07, p = 0.283). Results from the linear regression after transformation are presented in Table 3 using the reciprocal RT variables.

**Table 3:** Linear regression models for reaction time and age.

Variable	R	R squared	F-value (p-value)	Regression coefficient*
**Reciprocal RT hands**				
Male	.712	.504	145.1 (<0.001)	-0.12 (95% CI: -0.14;-0.10)
Female	.660	.433	159.8 (<0.001)	-0.11 (95% CI: -0.13;-0.09)
**Reciprocal RT feet**				
Male	.791	.626	234.1 (<0.001)	-0.11 (95% CI: -0.12;-0.09)
Female	.739	.546	246.2 (<0.001)	-0.09 (95% CI: -0.10;-0.08)

*regression coefficient and their confidence intervals are multiplied by 10,000 for the sake of visualization

**Fig 1:**
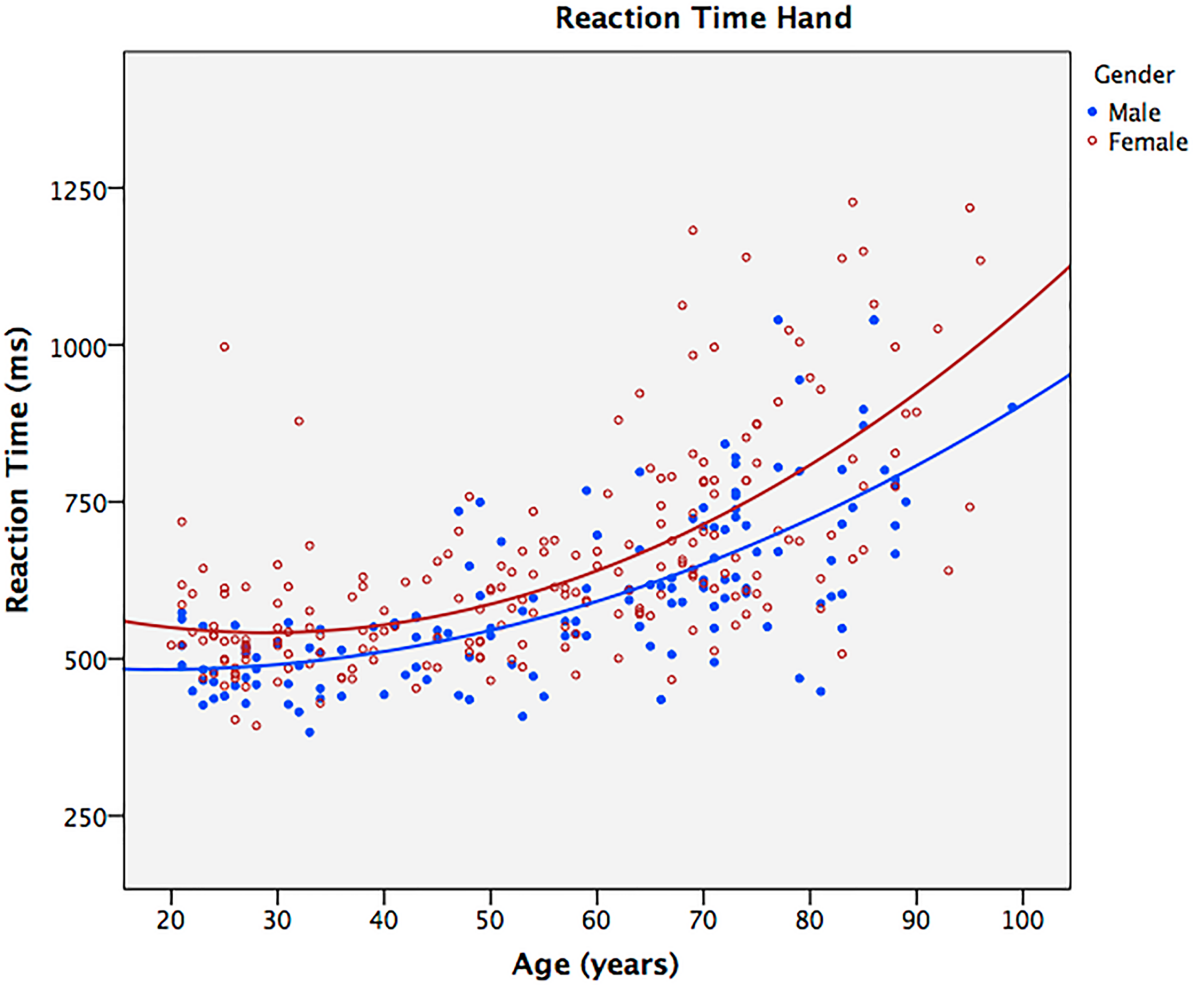
Reaction time hands: data points and quadratic models.

**Fig 2:**
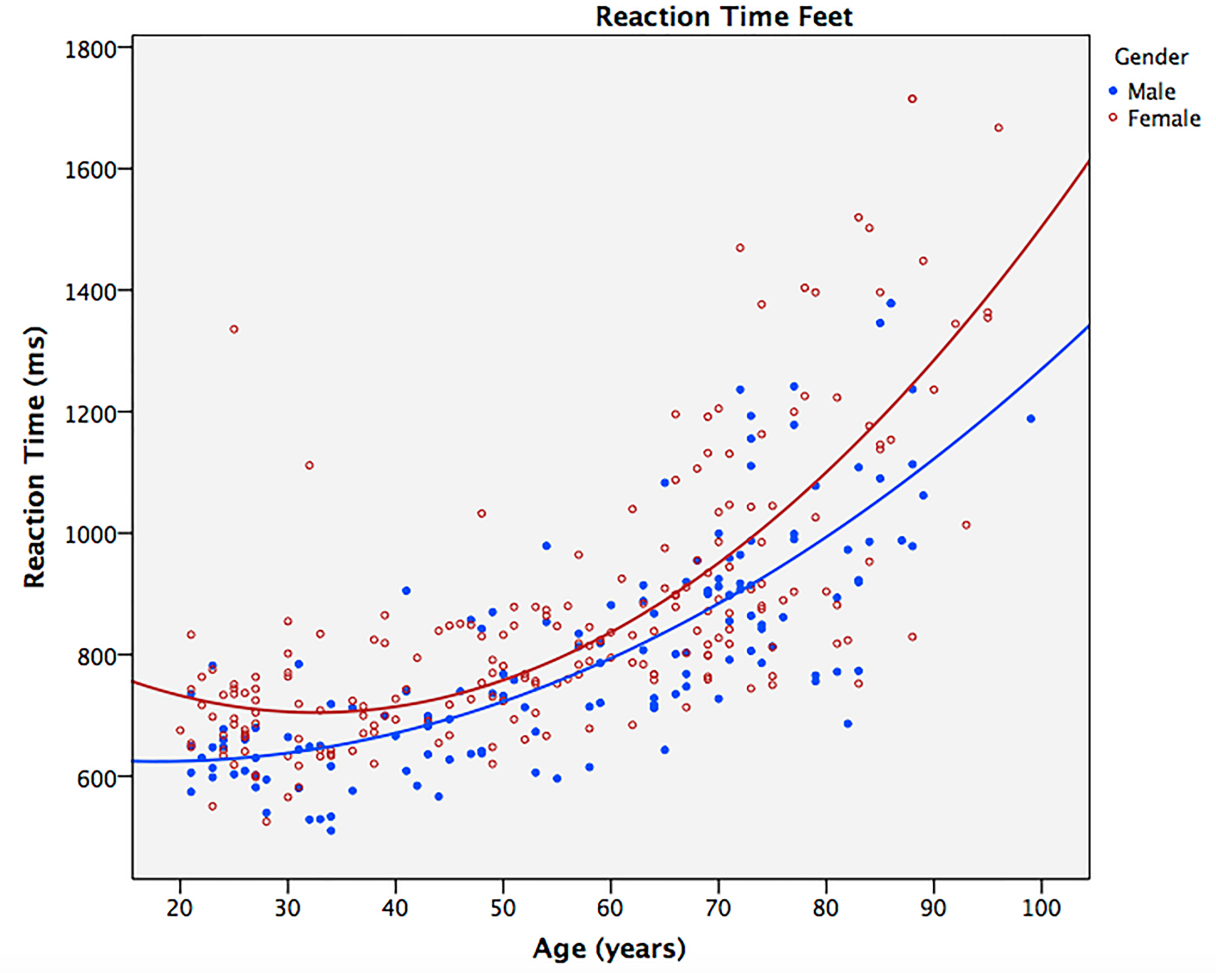
Reaction time feet: data points and quadratic models.

Without transformation, there was evidence of non-linearity, especially for the RT feet variables, as well as some indications of heteroscedastisity. As indicated by the negative regression coefficients, there was a statistically significant positive relationship with RT and age. On average, age could account for 47% and 59% of the variation in reciprocal RT for hands and feet, respectively. As indicated by the F-values, predictions of reciprocal RT based on age were superior for RT feet compared to RT hands. The hierarchical multiple regression confirmed a statistically significant non-linear relationship between RT and age. For RT hands, the quadratic model gave a significant R^2^ change of 3.2% (F change = 9.1, p < 0.001) and 5.6% (F change = 21.1, p < 0.001) for males and females, respectively. For RT feet, the R^2^ change was 3.6% (F change = 13.0, p < 0.001) and 9.4% (F change = 44.3, p < 0.001) for males and females, respectively. All beta coefficients for age squared were positive, meaning that the rate of increased RT increases with age. The quadratic models together with the raw data are presented graphically as the curved line in Figs 1 and 2.

## Discussion

This study reported reference data on RT and investigated its relationship with age using a standard WBB. The main findings for both feet and hands were; (1) increased mean RT and variation with increasing age, (2) males had faster RT compared to females, and (3) a significant increase in the rate of increase in RT with age.

It is well established that RT and its variability increases with age [18,35–37]. Less is known about sex differences. In our study, we found a consistent lower RT for males compared to females in every age group. The difference between male and female was statistically significant for both hands and feet. In the United Kingdom Health and Lifestyle Survey (HALS), using RT data from over 7,000 participants, consistent sex differences for simple and four-choice RT were also reported [37]. The method used in our study was a two-choice RT, meaning that the participant not only had to respond to a particular stimulus (simple RT), but also had to respond in one of two ways depending on the stimulus. This required additional information-processing in the central nervous system compared to simple RT. The sex differences for mean choice RT are reported to be the weakest and most variable. Being more extensively studied than simple RT, it could explain why studies with smaller samples and incomplete age coverage could yield ambiguous results [37]. However, in general, the present method and study protocol has identified these differences, and further supports the results from larger and more robust studies [38].

The HALS study also reported that mean RT increases more rapidly at older ages for females compared to males. By looking at the increased curvature in our quadratic models, this phenomenon was also apparent in our study. A possible cause for this accelerated increase in RT, albeit not the only one, is that older men represent a healthier subset compared to women due to sex differences in survival. However, larger cohort studies have only found negligible association between RT measures and medical or lifestyle factors [18], and no clear trend could be drawn from our anthropometric data either (Table 1). However, it should be noted that we used a rather crude measure for physical activity (four groups) which placed most of our individuals in group 2 or 3. Also, the age difference in activity level was masked by the fact that the older participants reported their total amount of physical activity as leisure time while young participants reported leisure time and working hours separately. Still, in contrast to lifestyle factors, biological factors such as vision, grip strength, and forced expiratory volume at one second, have shown to be more important for explaining age differences in RT performance [18].

Concerning our third main finding, the HALS study identified a similar quadratic curvilinear relationship between age and choice RT for both genders. This was statistically tested and confirmed with hierarchical multiple regression. In our linear regression models, age could account for more than half of the RT variation in males and approximately half of the RT variation in females. Interestingly, the models also showed that RT feet were better predicted with age than RT hands. Furthermore, HALS reported that their mean four-choice RT slows throughout adulthood, whereas simple mean RT barely slows until around 50 years of age. It is of particular interest that our findings, using a two-choice RT test, were something in between. We found that the average slowing of RT was evident after around 40 years of age for feet, whereas the slowing of RT was continuous throughout adulthood for hands. Since RT depends on the complexity of the task [39], and although both require response selection, the differences may in part be because two-choice RT is simpler to perform than a four-choice RT. In short, the main findings from our cross-sectional study using WBB were consistent with larger studies on RT and age [38].

Since the RT test used in our study has been shown to have good reproducibility with little or no learning across test sessions [24], the reference data and percentiles provided in this study can be used to identify subjects with increased RT. This may have implications for further research and clinical work. As noted previously, RT is one of the documented risk factors for falling [8–10]. The RT test is essentially a speed test of the cognitive-motor interaction, which is a major component of the motor skills required for successful balance recovery [40]. Additionally, the WBB allows for reliable testing of balance [41] and strength [28,29], both of which are important risk factors for falling [3,5]. The reliable assessment of these risk factors has generally been restricted to specialized fall clinics due to the significant costs of the stationary laboratory equipment needed. However, WBB offers a low-cost, highly mobile, and objective global test for these different components of the cognitive-motor interaction. In this regard, it is interesting to note that a recent systematic review and meta-analysis found reactive and volitional stepping interventions to reduce falls among olders adults by approximately 50% [42]. These encouraging results are significantly better than those observed with general exercise interventions [43,44]. The authors attribute this effect to the significant improvements in simple and choice RT, gait, and balance found with stepping interventions [42]. In short, the WBB offers a reliable assessment of the important components of the cognitive-motor interaction needed to prevent falls, and offers a novel opportunity to implement these tests in community-dwelling adults. However, more research is needed to confirm the relevance of these specific tests in fall risk assessments.

There are several strengths with the current study. Firstly, the data and method appear externally valid when compared to other larger studies on RT. Secondly, we used and reported relevant statistics, such as percentiles for further studies, and statistically tested for a significant non-linear relationship between age and RT. We also reported relevant anthropometric data for comparisons with other studies. Thirdly, the RT data were collected in the subjects’ home as a battery of three clinically relevant tests, which is similar to a real-life scenario of home-based fall-risk assessment. Finally, the data were averaged and collected for all age groups from six assessors with different educational background. However, our study also has significant limitations. Due to the non-random selection of participants we can not rule out the risk of selection bias. From the anthropometric data, we found some evidence of a comparatively younger representation in age groups 30–39 and 70–79. However, with the exception of females 70–79 for RTH-D, all age groups followed a similar trend for all four RT variables. Furthermore, all raters recruited members from different locations. For these reasons, selection bias may be minimal in the present study. Still, compared to epidemiological studies, our sample size was relatively small. We aimed for a healthy study-population, but did not include a standard clinical test for cognitive deficits. Thus, we can only say that the participants did not present with obvious cognitive deficits during data collection. How the data would look in a high-risk population for falling, or other relevant populations, is not known. Our percentiles are simply an approximation of the normal values, and not cut-offs for any particular risk factor. Further research is needed to investigate any predictive utility of RT using the WBB. Also, the informative value of this study to fall related measures is limited due to the restricted numbers of variables presented. Lastly, we made a crucial effort to harmonize our assessors for this study since RT assessments are susceptible to different experimental protocols and no studies have investigated the interrater reproducibility of our protocol. However, other researchers and assessors, even while trying to mimic our protocol, may end up with a slightly different protocol and resulting RT data. More studies are needed to detect, and preferably minimize, such potential protocol bias.

## Conclusion

In this study, we reported reference data with percentiles on a new promising method for reliable RT testing in a healthy population of 354 subjects. Our data using WBB found similar age and gender effects on RT as those previously reported in trials using conventional measurements. These include increased RT mean and variation with increasing age, lower RT among males compared to females, and a significant non-linear relationship between RT and age. The aforementioned data can be used to identify those with high aRT, and has the potential to improve the accuracy of fall risk assessments in community-dwelling older adults. The WBB has the potential to be a multimodal test and intervention instrument for clinically relevant parameters, previously only available at specialized fall clinics.

## Supporting information

S1 File Raw data. (XLSX)

S2 File Histograms. (PDF)

## References

[1] WHO. Globel Health and Aging [Internet]. 2011 [cited 2017 May 8]. Available from: http://www.who.int/ageing/publications/global_health/en/

[2] Christensen K, Doblhammer G, Rau R, Vaupel JW. Ageing populations: the challenges ahead. Lancet [Internet]. 2009 10 3 [cited 2017 Apr 30];374(9696):1196–208. Available from: http://www.ncbi.nlm.nih.gov/pubmed/19801098 doi: 10.1016/S0140-6736(09)61460-4 19801098 10.1016/S0140-6736(09)61460-4 PMC2810516

[3] Rubenstein LZ. Falls in older people: epidemiology, risk factors and strategies for prevention. Age Ageing [Internet]. 2006 9 [cited 2014 Jul 10];35 Suppl 2:ii37–ii41. Available from: http://www.ncbi.nlm.nih.gov/pubmed/16926202 16926202 10.1093/ageing/afl084

[4] Kannus P, Sievänen H, Palvanen M, Järvinen T, Parkkari J. Prevention of falls and consequent injuries in elderly people. Lancet [Internet]. 2005 11 26 [cited 2017 May 8];366(9500):1885–93. Available from: http://www.ncbi.nlm.nih.gov/pubmed/16310556 doi: 10.1016/S0140-6736(05)67604-0 16310556 10.1016/S0140-6736(05)67604-0

[5] Wang J, Chen Z, Song Y. Falls in aged people of the Chinese mainland: epidemiology, risk factors and clinical strategies. Ageing Res Rev [Internet]. 2010 11 [cited 2015 Dec 1];9 Suppl 1:S13–7. Available from: http://www.ncbi.nlm.nih.gov/pubmed/20667514 20667514 10.1016/j.arr.2010.07.002

[6] Boehm J, Franklin RC, King JC. Falls in rural and remote community dwelling older adults: A review of the literature. Aust J Rural Health [Internet]. 2014 8 [cited 2017 Feb 12];22(4):146–55. Available from: http://doi.wiley.com/10.1111/ajr.12114 25123617 10.1111/ajr.12114

[7] Campbell AJ, Robertson MC. Rethinking individual and community fall prevention strategies: a meta-regression comparing single and multifactorial interventions. Age Ageing [Internet]. 2007 11 1 [cited 2017 May 8];36(6):656–62. Available from: https://academic.oup.com/ageing/article-lookup/doi/10.1093/ageing/afm122 18056731 10.1093/ageing/afm122

[8] Lord SR, Ward JA, Williams P, Anstey KJ. Physiological factors associated with falls in older community-dwelling women. J Am Geriatr Soc [Internet]. 1994 10 [cited 2015 Dec 11];42(10):1110–7. Available from: http://www.ncbi.nlm.nih.gov/pubmed/7930338 7930338 10.1111/j.1532-5415.1994.tb06218.x

[9] Lord SR, Clark RD. Simple physiological and clinical tests for the accurate prediction of falling in older people. Gerontology [Internet]. 1996 1 [cited 2016 Jan 29];42(4):199–203. Available from: http://www.ncbi.nlm.nih.gov/pubmed/8832267 8832267 10.1159/000213793

[10] Maver SL, Dodd K, Menz H. Lower limb reaction time discriminates between multiple and single fallers. Physiother Theory Pract [Internet]. 2011 7 [cited 2016 Aug 24];27(5):329–36. Available from: http://www.ncbi.nlm.nih.gov/pubmed/20795877 doi: 10.3109/09593985.2010.510551 20795877 10.3109/09593985.2010.510551

[11] Davis JC, Best JR, Khan KM, Dian L, Delbaere K, Hsu CL, et al Slow Processing Speed Predicts Falls in Older Adults With a Falls History: 1-Year Prospective Cohort Study. J Am Geriatr Soc [Internet]. 2017 5;65(5):916–923. Available from: https://www.ncbi.nlm.nih.gov/pubmed/28390178 doi: 10.1111/jgs.14830 28390178 10.1111/jgs.14830

[12] Chen TY, Peronto CL, Edwards JD. Cognitive Function as a Prospective Predictor of Falls. J Gerontol B Psychol Sci Soc Sci [Internet]. 2012 11;67(6):720–728. Available from: https://www.ncbi.nlm.nih.gov/pubmed/22865822 doi: 10.1093/geronb/gbs052 22865822 10.1093/geronb/gbs052 PMC3636670

[13] Graveson J, Bauermeister S, McKeown D, Bunce D. Intraindividual Reaction Time Variability, Falls and Gait in Old Age: A Systematic Review. J Gerontol B Psychol Sci Soc Sci [Internet]. 2016 9;71(5):857–64. Available from: https://www.ncbi.nlm.nih.gov/pubmed/25969471 doi: 10.1093/geronb/gbv027 25969471 10.1093/geronb/gbv027

[14] Bisson E, Contant B, Sveistrup H, Lajoie Y. Functional Balance and Dual-Task Reaction Times in Older Adults Are Improved by Virtual Reality and Biofeedback Training. CyberPsychology Behav [Internet]. 2007 2 [cited 2017 Feb 12];10(1):16–23. Available from: http://www.ncbi.nlm.nih.gov/pubmed/17305444 10.1089/cpb.2006.9997 17305444

[15] Zijlstra A, Mancini M, Chiari L, Zijlstra W. Biofeedback for training balance and mobility tasks in older populations: a systematic review. J Neuroeng Rehabil [Internet]. 2010 12 9 [cited 2017 Feb 12];7(1):58 Available from: http://www.ncbi.nlm.nih.gov/pubmed/21143921 21143921 10.1186/1743-0003-7-58 PMC3019192

[16] Crabtree DA, Antrim LR. Guidelines for measuring reaction time. Percept Mot Skills [Internet]. 1988 4 [cited 2016 Aug 24];66(2):363–70. Available from: http://www.ncbi.nlm.nih.gov/pubmed/3399312 doi: 10.2466/pms.1988.66.2.363 3399312 10.2466/pms.1988.66.2.363

[17] Darbutas T, Juodžbalienė V, Skurvydas A, Kriščiūnas A. Dependence of reaction time and movement speed on task complexity and age. Medicina (Kaunas) [Internet]. 2013 [cited 2016 Sep 19];49(1):18–22. Available from: http://www.ncbi.nlm.nih.gov/pubmed/23652713 23652713

[18] Anstey KJ, Dear K, Christensen H, Jorm AF. Biomarkers, health, lifestyle, and demographic variables as correlates of reaction time performance in early, middle, and late adulthood. Q J Exp Psychol Sect A [Internet]. Psychology Press; 2005 1 [cited 2017 Feb 12];58(1):5–21. Available from: http://www.tandfonline.com/doi/abs/10.1080/02724980443000232 15881288 10.1080/02724980443000232

[19] Lajoie Y, Gallagher SP. Predicting falls within the elderly community: comparison of postural sway, reaction time, the Berg balance scale and the Activities-specific Balance Confidence (ABC) scale for comparing fallers and non-fallers. Arch Gerontol Geriatr [Internet]. [cited 2016 Aug 24];38(1):11–26. Available from: http://www.ncbi.nlm.nih.gov/pubmed/14599700 14599700 10.1016/s0167-4943(03)00082-7

[20] Jorgensen MG, Paramanathan S, Ryg J, Masud T, Andersen S. Novel use of the Nintendo Wii board as a measure of reaction time: a study of reproducibility in older and younger adults. BMC Geriatr [Internet]. 2015 1 [cited 2015 Sep 8];15:80 Available from: http://www.pubmedcentral.nih.gov/articlerender.fcgi?artid=4496937&tool=pmcentrez&rendertype=abstract doi: 10.1186/s12877-015-0080-6 26155934 10.1186/s12877-015-0080-6 PMC4496937

[21] Mercer VS, Hankins CC, Spinks AJ, Tedder DD. Reliability and validity of a clinical test of reaction time in older adults. J Geriatr Phys Ther [Internet]. 2009 [cited 2016 Sep 19];32(3):103–10. Available from: http://www.ncbi.nlm.nih.gov/pubmed/20128334 20128334 10.1519/00139143-200932030-00004

[22] Yamada M, Aoyama T, Nakamura M, Tanaka B, Nagai K, Tatematsu N, et al The Reliability and Preliminary Validity of Game-Based Fall Risk Assessment in Community-Dwelling Older Adults. Geriatr Nurs (Minneap) [Internet]. 2011 5 [cited 2017 Feb 12];32(3):188–94. Available from: http://www.ncbi.nlm.nih.gov/pubmed/21501899 10.1016/j.gerinurse.2011.02.002 21501899

[23] Fysiometer. Fysiometer [Internet]. Available from: http://fysiometer.dk/

[24] Jorgensen MG, Paramanathan S, Ryg J, Masud T, Andersen S. Novel use of the Nintendo Wii board as a measure of reaction time: a study of reproducibility in older and younger adults. BMC Geriatr [Internet]. 2015 [cited 2016 Aug 23];15:80 Available from: http://www.ncbi.nlm.nih.gov/pubmed/26155934 doi: 10.1186/s12877-015-0080-6 26155934 10.1186/s12877-015-0080-6 PMC4496937

[25] Blomkvist AW, Andersen S, de Bruin ED, Jorgensen MG. Isometric hand grip strength measured by the Nintendo Wii Balance Board—a reliable new method. BMC Musculoskelet Disord [Internet]. 2016 1 [cited 2016 Feb 17];17(1):56 Available from: http://www.pubmedcentral.nih.gov/articlerender.fcgi?artid=4739099&tool=pmcentrez&rendertype=abstract 26842966 10.1186/s12891-016-0907-0 PMC4739099

[26] Clark RA, Bryant AL, Pua Y, McCrory P, Bennell K, Hunt M. Validity and reliability of the Nintendo Wii Balance Board for assessment of standing balance. Gait Posture [Internet]. 2010 3 [cited 2014 Jul 13];31(3):307–10. Available from: http://www.ncbi.nlm.nih.gov/pubmed/20005112 doi: 10.1016/j.gaitpost.2009.11.012 20005112 10.1016/j.gaitpost.2009.11.012

[27] Larsen LR, Jørgensen MG, Junge T, Juul-Kristensen B, Wedderkopp N. Field assessment of balance in 10 to 14 year old children, reproducibility and validity of the Nintendo Wii board. BMC Pediatr [Internet]. 2014 12 10 [cited 2017 Feb 12];14(1):144 Available from: http://www.ncbi.nlm.nih.gov/pubmed/24913461 24913461 10.1186/1471-2431-14-144 PMC4057805

[28] Blomkvist AW, Andersen S, de Bruin E, Jorgensen MG. Unilateral lower limb strength assessed using the Nintendo Wii Balance Board: a simple and reliable method. Aging Clin Exp Res [Internet]. Springer International Publishing; 2016 12 19 [cited 2016 Dec 20];1–8. Available from: http://link.springer.com/10.1007/s40520-016-0692-5 10.1007/s40520-016-0692-5 27995527

[29] Gronbech Jorgensen M, Andersen S, Ryg J, Masud T. Novel Use of the Nintendo Wii Board for Measuring Isometric Lower Limb Strength: A Reproducible and Valid Method in Older Adults. PLoS One [Internet]. Public Library of Science; 2015 1 [cited 2016 Jan 29];10(10):e0138660 Available from: http://journals.plos.org/plosone/article?id=10.1371/journal.pone.0138660 10.1371/journal.pone.0138660 PMC4596703 26444554

[30] Laufer Y, Dar G, Kodesh E. Does a Wii-based exercise program enhance balance control of independently functioning older adults? A systematic review. Clin Interv Aging [Internet]. 2014 1 [cited 2015 Jun 23];9:1803–13. Available from: http://www.pubmedcentral.nih.gov/articlerender.fcgi?artid=4211857&tool=pmcentrez&rendertype=abstract doi: 10.2147/CIA.S69673 25364238 10.2147/CIA.S69673 PMC4211857

[31] Schnohr P. Physical activity in leisure time: impact on mortality. Risks and benefits. Dan Med J [Internet]. 2009;56(1):40–71. Available from: https://www.ncbi.nlm.nih.gov/pubmed/?term=P.+Schnohr%2C+“Physical+activity+in+leisure+time%3A+impact+on+mortality.+Risks+and+benefits 19232166

[32] Jørgensen MG. Assessment of postural balance in community-dwelling older adults. Dan Med J [Internet]. 2014;61(1):B4775 Available from: http://www.ncbi.nlm.nih.gov/pubmed/24393594 24393594

[33] Hoaglin D, Iglewicz B. Fine-Tuning Some Resistant Rules for Outlier Labeling. J Am Stat Assoc. 1987;82(400):1147–9.

[34] Field A. Discovering Statistics Using IBM SPSS Statistics 4th editio SAGE Publications Ltd; 2013.

[35] Fozard JL, Vercryssen M, Reynolds SL, Hancock PA, Quilter RE. Age differences and changes in reaction time: the Baltimore Longitudinal Study of Aging. J Gerontol [Internet]. 1994 7 [cited 2017 Mar 23];49(4):P179–89. Available from: http://www.ncbi.nlm.nih.gov/pubmed/8014399 8014399 10.1093/geronj/49.4.p179

[36] Deary IJ, Der G. Reaction Time, Age, and Cognitive Ability: Longitudinal Findings from Age 16 to 63 Years in Representative Population Samples. Aging, Neuropsychol Cogn [Internet]. Taylor & Francis Group; 2005 6 [cited 2017 Mar 23];12(2):187–215. Available from: http://www.tandfonline.com/doi/abs/10.1080/13825580590969235

[37] Der G, Deary IJ. Age and sex differences in reaction time in adulthood: Results from the United Kingdom Health and Lifestyle Survey. Psychol Aging [Internet]. 2006 [cited 2016 Nov 2];21(1):62–73. Available from: http://doi.apa.org/getdoi.cfm?doi=10.1037/0882-7974.21.1.62 16594792 10.1037/0882-7974.21.1.62

[38] Dykiert D, Der G, Starr JM, Deary IJ. Age Differences in Intra-Individual Variability in Simple and Choice Reaction Time: Systematic Review and Meta-Analysis. Bayer A, editor. PLoS One [Internet]. 2012 10 11 [cited 2017 Mar 23];7(10):e45759 Available from: http://www.ncbi.nlm.nih.gov/pubmed/23071524 doi: 10.1371/journal.pone.0045759 23071524 10.1371/journal.pone.0045759 PMC3469552

[39] Darbutas T, Juodžbalienė V, Skurvydas A, Kriščiūnas A. Dependence of reaction time and movement speed on task complexity and age. Medicina (Kaunas) [Internet]. 2013 [cited 2017 Mar 23];49(1):18–22. Available from: http://www.ncbi.nlm.nih.gov/pubmed/23652713 23652713

[40] Pijnappels M, Bobbert MF, van Dieën JH. How early reactions in the support limb contribute to balance recovery after tripping. J Biomech [Internet]. 2005 3 [cited 2017 Apr 21];38(3):627–34. Available from: http://www.ncbi.nlm.nih.gov/pubmed/15652564 doi: 10.1016/j.jbiomech.2004.03.029 15652564 10.1016/j.jbiomech.2004.03.029

[41] Jorgensen MG, Laessoe U, Hendriksen C, Nielsen OBF, Aagaard P. Intrarater reproducibility and validity of Nintendo Wii balance testing in community-dwelling older adults. J Aging Phys Act. 2014;22(2):269–75. doi: 10.1123/japa.2012-0310 23752090 10.1123/japa.2012-0310

[42] Okubo Y, Schoene D, Lord SR. Step training improves reaction time, gait and balance and reduces falls in older people: a systematic review and meta-analysis. Br J Sports Med [Internet]. 2017 4 [cited 2017 Apr 21];51(7):586–93. Available from: http://bjsm.bmj.com/lookup/doi/10.1136/bjsports-2015-095452 26746905 10.1136/bjsports-2015-095452

[43] Sherrington C, Tiedemann A, Fairhall N, Close JCT, Lord SR. Exercise to prevent falls in older adults: an updated meta-analysis and best practice recommendations. N S W Public Health Bull [Internet]. 2011 6 [cited 2017 Apr 21];22(4):78 Available from: http://www.ncbi.nlm.nih.gov/pubmed/21632004 21632004 10.1071/NB10056

[44] Gillespie LD, Robertson MC, Gillespie WJ, Sherrington C, Gates S, Clemson LM, et al Interventions for preventing falls in older people living in the community In: Gillespie LD, editor. Cochrane Database of Systematic Reviews [Internet]. Chichester, UK: John Wiley & Sons, Ltd; 2012 [cited 2017 Apr 21]. p. CD007146 Available from: http://www.ncbi.nlm.nih.gov/pubmed/22972103 doi: 10.1002/14651858.CD007146.pub3 10.1002/14651858.CD007146.pub3 PMC8095069 22972103

