# Supplementary figures and images for: Reference data on reaction time and aging using the Nintendo Wii Balance Board: A cross-sectional study of 354 subjects from 20 to 99 years of age
Source: PLoS One. 2017 Dec 29;12(12):e0189598. doi: 10.1371/journal.pone.0189598 (PMC5747451; doi:10.1371/journal.pone.0189598)

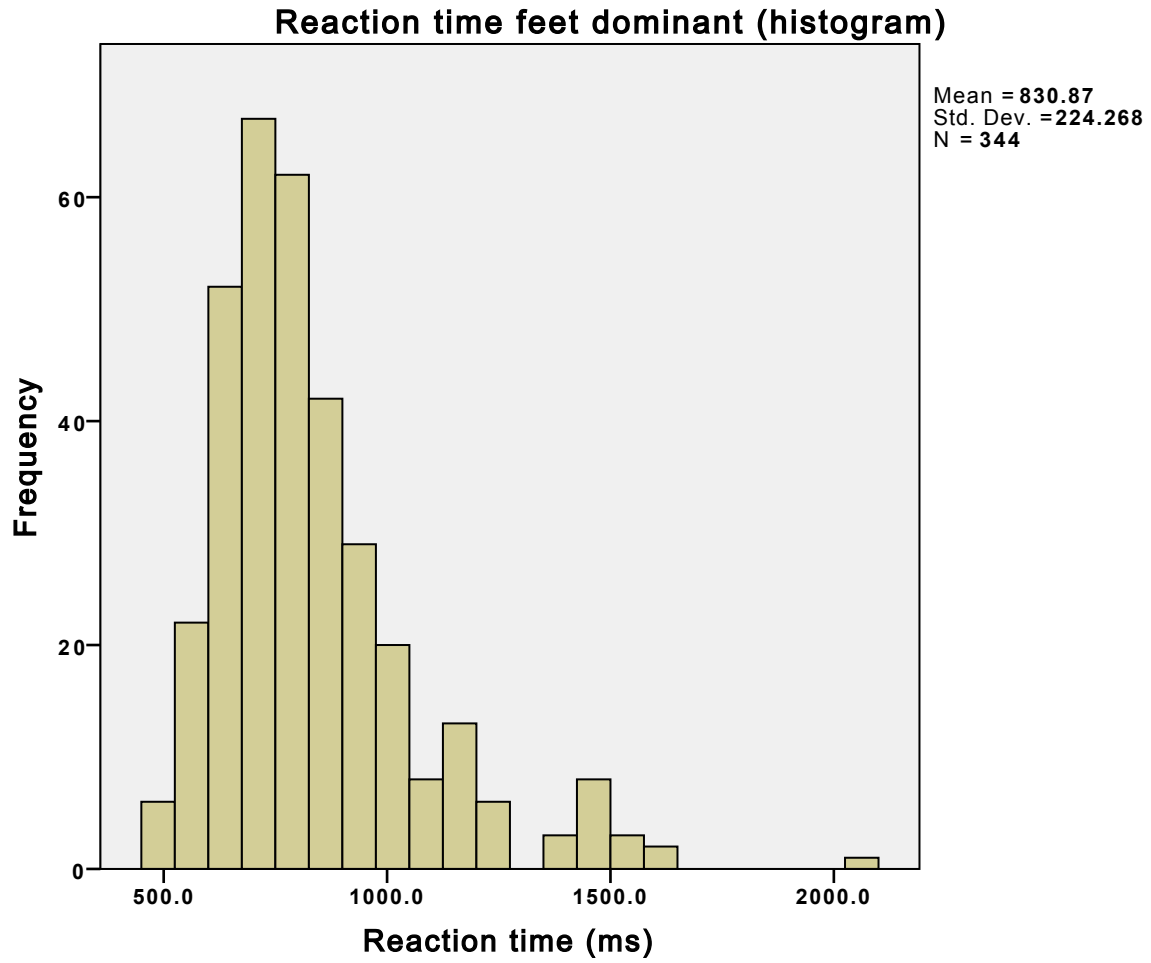

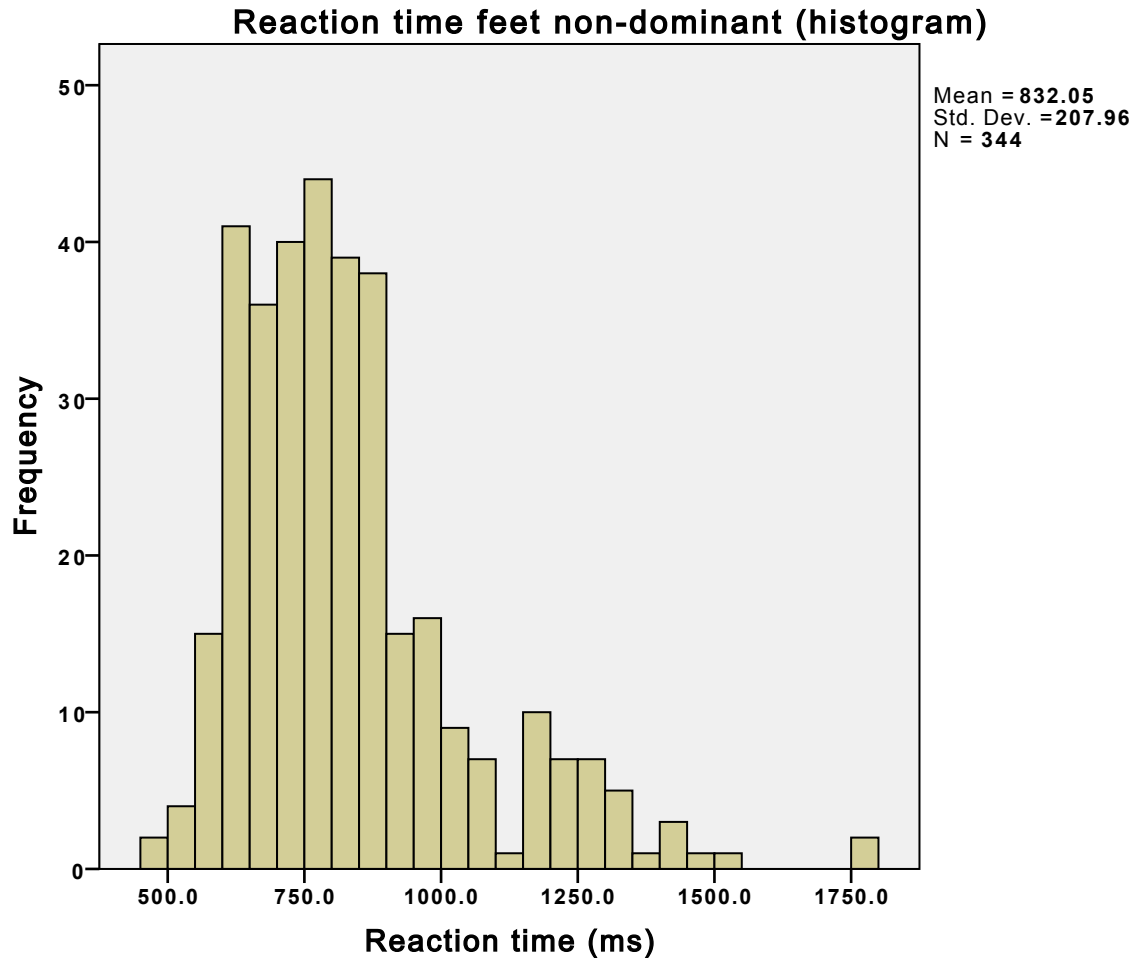

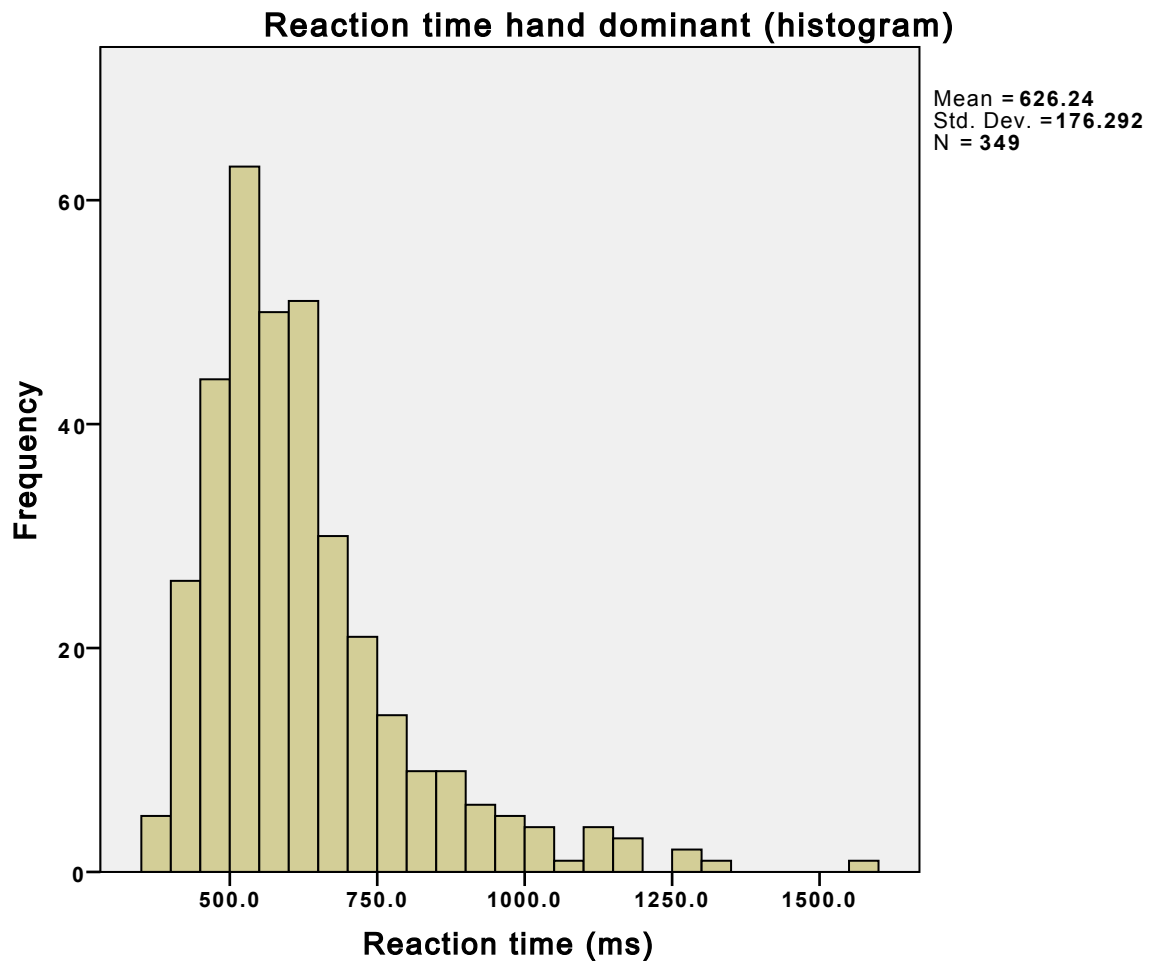

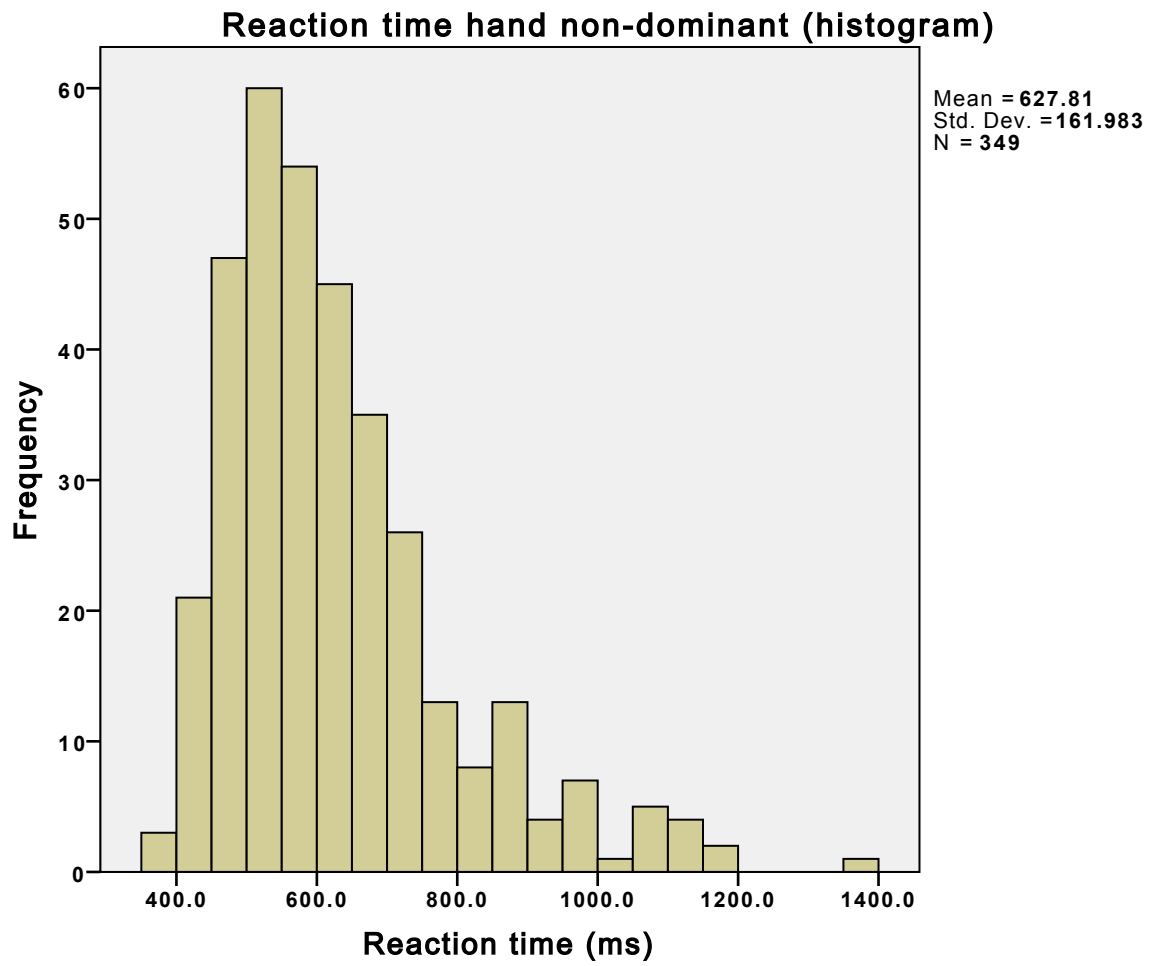

Supplement: S2 File — (PDF) [file pone.0189598.s002.pdf]
